# Specific immunotherapy ameliorates ulcerative colitis

**DOI:** 10.1186/s13223-016-0142-0

**Published:** 2016-08-05

**Authors:** Min Cai, Lu Zeng, Lin-Jing Li, Li-Hua Mo, Rui-Di Xie, Bai-Sui Feng, Peng-Yuan Zheng, Zhi-Gang Liu, Zhan-Ju Liu, Ping-Chang Yang

**Affiliations:** 1 Department of Gastroenterology, Shanghai Tenth People’s Hospital of Tongji University, Shanghai, China; 2 The Center of Allergy & Immunology, Shenzhen University School of Medicine, Shenzhen, China; 3 Department of Gastroenterology, The Fifth Affiliated Hospital of Zhengzhou University, Zhengzhou, China

**Keywords:** Food allergy, Intestine, Inflammation, B lymphocyte, Immunoglobulin E

## Abstract

**Background:**

Hypersensitivity reaction to certain allergens plays a role in the pathogenesis of inflammatory bowel disease (IBD). This study aims to observe the effect of specific immunotherapy in a group of IBD patients.

**Methods:**

Patients with both ulcerative colitis (UC) and food allergy were recruited into this study. Food allergy was diagnosed by skin prick test and serum specific IgE. The patients were treated with specific immunotherapy (SIT) and *Clostridium butyricum* (CB) capsules.

**Results:**

After treating with SIT and CB, the clinical symptoms of UC were markedly suppressed as shown by reduced truncated Mayo scores and medication scores. The serum levels of specific IgE, interleukin (IL)-4 and tumor necrosis factor (TNF)-α were also suppressed. Treating with SIT alone or CB alone did not show appreciable improvement of the clinical symptoms of UC.

**Conclusions:**

UC with food allergy can be ameliorated by administration with SIT and butyrate-production probiotics.

## Background

Inflammatory bowel disease (IBD) is a kind of chronic inflammatory bowel disease, including Crohn’s disease (CD) and chronic nonspecific ulcerative colitis (UC). The pathogenesis of IBD is unknown. Numerous reports implicate that immune abnormalities, infection or genetic factors contribute to IBD [1]. The colon is the most common location of IBD. Because of the high density of microbes in the colon and the hyper-reactivity of the immune system in IBD patients, it is assumed that over-reaction to microbes or their products is a major causative factor of IBD. However, so far we do not have satisfactory therapies for IBD.

It has been noted that food allergy is associated with the pathogenesis of IBD [2, 3]. Food allergy is an adverse reaction of the immune system to innocent nutritional elements, such as eggs, fish, cow milk, etc. The pathologic feature of food allergy is that antigen-specific IgE forms a complex with the high affinity receptor of IgE on the surface of mast cells, which makes mast cells sensitized. Upon re-exposure to the specific antigen, the sensitized mast cells can be activated and release a series of chemical mediators, such as histamine, leukotrienes, tryptase, tumor necrosis factor (TNF)-α, etc. These chemical mediators are capable of inducing inflammation in the local tissue.

The current therapeutic remedies of IBD include drug treatment (such as immunomodulators and biologic therapies); the formation of intestinal stenosis needs surgical treatment. Probiotic treatment is also a therapeutic option [4]. In fact, the current therapies for IBD have important limitations currently. Aminosalicylates 2–4 are commonly used for UC, but the efficacy is modest. Steroids are effective, but cause severe complications. Anti-tumor necrosis factor (TNF)-α antibody is effective in some IBD patients [5], but may cause serious infection in some predispose patients [6]. Therefore, new treatment remedies are needed. The therapies for food allergy are also limited. Avoidance of the offending foods is the major method. Another remedy for food allergy treatment is the allergen specific immunotherapy (SIT), which is recommended to be a specific remedy for food allergy by the World Health Organization [7].

Published data indicate that probiotics have therapeutic effects on immune disorders [8]. Probiotics are bacteria that can provide healthy benefit when consumed. The benefits of probiotics include the decrease of pathogenic microorganisms in the intestine, regulation of gastrointestinal function, to strengthen the function of immune system, to improve the skin’s function, etc. [9]. Administration of probiotics has got promising therapeutic effects [10]. Probiotics also show benefit in the improvement of IBD symptoms while the methodology in their administration needs to be further investigated [4].

Based on the information above, we hypothesize that SIT can be a specific remedy for IBD with food allergy. In this study, we treated a group of UC/food allergy patients with SIT and *Clostridium butyricum* (CB). The results showed a satisfactory alleviation on IBD symptoms.

## Methods

### Ethics approval and consent to participate

This study was approved by the Human Research Ethic Committee at Tongji University and Zhengzhou University. The experiments were performed in accordance with the approved guidelines. An informed, written consent was obtained from each patient. The project was registered as a clinical study at the Clinical Practice Registration Bureau of Shanghai, China (CPRB#2013086; March 21, 2013).

### UC patients with food allergy

Patients with both UC and food allergy were diagnosed by physicians at our clinic following the published procedures [11, 12]. The UC-related information is presented in Table 1. The diagnosis of food allergy was based on the disease history, skin prick test (SPT) (Table 2) with food antigen extracts and serum specific IgE (>0.35 IU/ml).

**Table 1:** Demographic data

Characteristic	Placebo	SIT	CB	SIT/CB
	(N = 22)	(N = 23)	(N = 25)	(N = 26)
Age (years)	43.5 ± 11.2	41.8 ± 12.5	42.2 ± 8.6	43.3 ± 10.7
Male-no. (%)	12 (54.5)	11 (47.8)	14 (56)	14 (53.8)
Body weight (kg)	62.3 ± 11.5	61.4 ± 12.6	60.8 ± 14.1	61.8 ± 10.9
Current smoker-no. (%)	4 (18.2)	3 (13)	2 (8)	3 (11.5)
Duration of disease (years)	6.2 ± 5.2	5.8 ± 6.1	6.3 ± 4.9	6.3 ± 5.4
Mayo clinic score (MCS)	8.5 ± 1.2	8.6 ± 1.8	8.6 ± 1.1	8.6 ± 1.6
Partial MCS	6.1 ± 1.1	6.0 ± 1.4	6.1 ± 1.4	6.0 ± 1.5
IBDQ score	125 ± 36	126 ± 32	123 ± 33	125 ± 30
Fecal calprotectin (mg/g)	1.2 ± 1.1	1.1 ± 0.9	1.2 ± 0.8	1.1 ± 0.8
Site of disease-no. (%)				
Rectum and sigmoid colon	4 (18.2)	6 (27.3)	5 (20)	6 (23.1)
Left side of colon	6 (27.3)	6 (27.3)	8 (32)	6 (23.1)
Proximal colon	7 (31.8)	5 (21.7)	3 (8)	5 (19.2)
All of the colon	5 (22.7)	6 (27.3)	9 (36)	9 (34.6)
Medication-no. (%)				
Steroids	3 (13.6)	4 (17.4)	5 (20)	7 (26.9)
Immunosuppressor	7 (31.8)	5 (21.7)	7 (28)	6 (23.1)
Steroids/immunosuppressor	6 (27.3)	5 (21.7)	7 (28)	7 (26.9)
No medication	6 (27.3)	9 (39.1)	6 (24)	6 (23.1)
Fecal calprotectin				
Before	255.6 (38.6)	266.8 (42.5)	249.5 (41.3)	253.8 (39.5)
After	244.8 (36.8)	258.3 (41.7)	241.5 (39.5)	68.3 (8.5)*
Hemoglobin (g/l)				
Before	122.5 ± 15.8	123.8 ± 17.6	125.6 ± 15.2	122.8 ± 14.5
After	124.3 ± 14.2	123.1 ± 13.6	125.2 ± 11.5	144.6 ± 11.1*
WBC (×10^−9^/l)				
Before	8.6 ± 3.2	8.6 ± 3.5	8.6 ± 3.2	8.5 ± 3.6
After	8.3 ± 2.5	8.2 ± 4.1	8.2 ± 2.4	6.1 ± 1.4*

*WBC* white blood cell count, *Before* before treatment, *After* after treatment

* p < 0.01, compared with the results before treatment in the same group

**Table 2:** Results of skin prick test

	Fish	Egg	Soy	Hazelnut	Shrimp	Walnut	Milk	Peanut
Patients	15/96	28/96	28/96	16/96	29/96	16/96	35/96	21/96
Percentage	(15.6)	(29.2)	(29.2)	(16.7)	(30.2)	(16.7)	(36.5)	(21.9)

Some patients were sensitized to more than one antigen. These are the most common food allergens in China. The number “96” is total IBD patients with food allergy

### Selection of non-IBD patients

To compare the incidence of food allergy between IBD patients and non-IBD patients, non-IBD subjects were also recruited with the criteria: no IBD disease history; no other organ diseases; no autoimmune diseases; no cancer. The diagnosis of food allergy in non-IBD subjects was in the same procedures as of IBD group.

### SPT

SPT was carried out in our clinic using commercial food extract reagents (Greer Company; Taibei, China). Food extracts included shrimp, eggs, walnuts, hazelnuts, soy, peanuts, fish and cow’s milk. Saline was used as a negative control. Histamine (1 mg/ml) was used as a positive control. SPT was defined as positive when the diameter of the wheal was 3 mm larger than a negative control at 15 min. The SPT results are presented in Table 2.

### UC clinical symptom assessment

The truncated Mayo score system (Table 3) was employed to assess the clinical UC symptoms (mainly including rectal bleeding and stool frequency), which were evaluated before and after the treatment.

**Table 3:** Truncated Mayo scores

Rectal bleeding scale
0. No blood seen
1. Streaks of blood with stool less than half of the time
2. Obvious blood with stool most of the time
3. Blood alone passed
Stool frequency scale
0. Normal stool frequency per day
1. 1–2 stools greater than normal per day
2. 3–4 stools greater than normal per day
3. ≥5 stools greater than normal per day

### Medication score assessment

If necessary, the patients were allowed to be treated with prednisone based on previous reports [13]. The medication scores (Table 4) were recorded for each patient 1 month before the therapy and the last month during the observation period.

**Table 4:** Medication scores

Mesalamine (g)	Scores	Prednisone (mg)	Scores
1	1	10	1
2	2	20	2
3	3	30	3
4	4	40	4

The dosage indicates daily dose

### Therapies

Patients were randomized to one of four groups: (1) SIT only (2) CB only, or (3) Combination SIT + CB, (4) Placebo. A 6-month SIT was carried out for each patient in SIT only group and the combination SIT + CB group. Briefly, UC patients were treated with the sensitized foods once a week for 10 weeks. The weekly increment dosage is presented in Table 5. From the 11th week to the end of the 6th month, the patients took the sensitized foods at tenfolds of the basic dose every 2 weeks. In the CB group, patients were prescribed with CB capsules (420 mg, two times a day) throughout the entire observation period. In the placebo group, patients were given saline instead of allergen vaccine, and the capsules contained vehicle instead of CB.

**Table 5:** Food antigen dosage

Fish	Egg	Soy	Hazelnut	Shrimp	Walnut	Milk	Peanut
3 g	1/10 egg	3 g	3 g	3 g	3 g	10 ml	3 g

### Collection of peripheral blood samples

Peripheral blood samples (20 ml per patient) were collected from each patient before and after the therapy. The sera were isolated by centrifugation (3000 rpm) for 10 min at 4 °C, and stored at −80 °C until use.

### Determination of serum Ig and cytokines

The serum levels of specific IgE and IgG4 levels were determined by the ImmunoCAP 250 system (Phadia, Uppsala, Sweden). The serum levels of IL-4, IL-17, IFN-γ and TNF-α were determined by enzyme-linked immunoassay (ELISA) with commercial reagent kits (R&D Systems, Minneapolis, MN) following the manufacturer’s instructions.

### Measurement of fecal calprotectin

Patients provided a stool sample for the measurement of calprotectin at the clinic visits. Fecal calprotectin was measured by ELISA with a reagent kit following the manufacturer’s instructions (Enke Biotech, Shenzhen, China). Laboratory personnel were blinded to the clinical data.

### Statistics

Data are presented as mean ± SD, or percentage. Difference between groups was determined by Student t test, or χ^2^ test. The significant criterion was set as p < 0.05.

## Results

### Patients

**Fig. 1:**
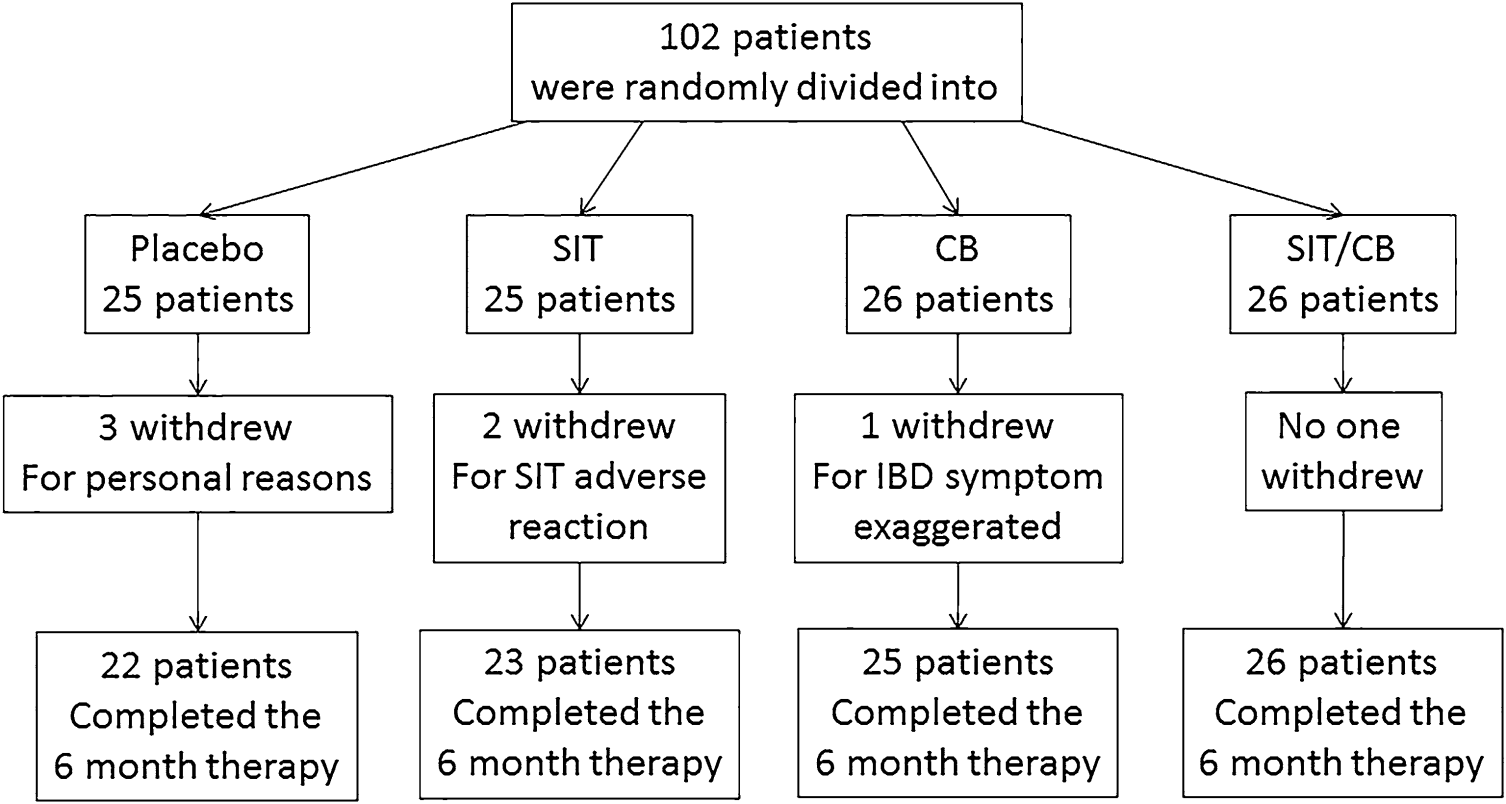
Patients completed the 6 month therapy. In total 102 UC patients were recruited into this study. The patients were randomly divided into four groups. Among them, two patients withdrew for SIT adverse reaction; one withdrew for IBD symptom exaggerated; three withdrew for personal reasons. Thus, 96 patients completed the 6 month therapy

Patient information is presented in Fig. 1. From 338 UC patients, 102 (30.17 %) UC patients were diagnosed food allergy and were recruited into this study. Among the patients, six withdrew from the study; thus, 96 patients completed the 6-month study. No patients were treated with SIT or CB or anti-TNF-α antibody previously. As shown by Table 1, no important difference in the demographic data between groups. On the other hand, we compared the incidence of food allergy between IBD patients and those without IBD. In total 342 non-IBD subjects (male: 170; female: 172; age: 42.6 ± 11.8 years old) were recruited, in which 8 (2.34 %) subjects had food allergy. The incidence of food allergy in non-IBD subjects was significantly less (p < 0.01) than IBD patients.

### Clinical outcomes of the therapy

**Fig. 2:**
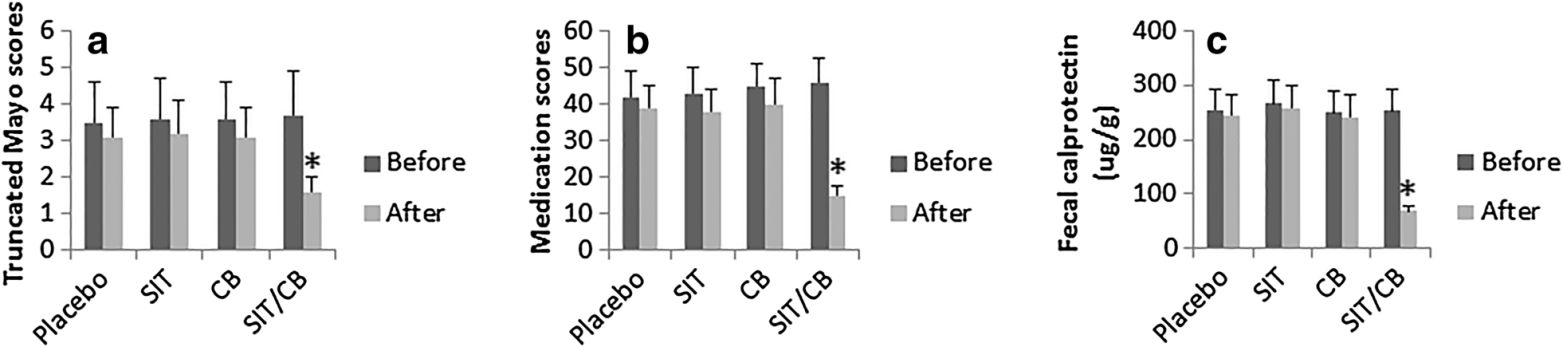
Clinical outcomes of the therapy. The *bars* indicate the outcomes of the therapy, including truncated Mayo scores (**a**), medication scores (**b**) and the levels of fecal calprotectin (**c**), which were recorded before and after completion of the therapy. *p < 0.01, compared with the placebo group. The patient numbers are the same as Fig. 1

As shown by Fig. 2a, the truncated Mayo scores reduced significantly in the SIT/CB group. In the patients treated with SIT alone or CB alone, the truncated Mayo scores were also reduced, but did not reach the significant criterion.

The medication scores were also significantly reduced in the patients treated with SIT/CB. Like the truncated Mayo scores, patients treated with SIT alone or CB alone did not show significant improvement of the medication scores (Fig. 2b).

We also assessed the changes of fecal calprotectin levels in the patients before and after the therapy. The results showed that the levels of fecal calprotectin were significantly reduced in patients treated with both SIT and CB, while those treated with either placebo, or SIT alone, or CB alone did not show significant changes of the fecal calprotectin (Fig. 2c).

### Effect of the therapy on serum IgE, IgG4 and CD4^+^ T cell cytokines

**Fig. 3:**
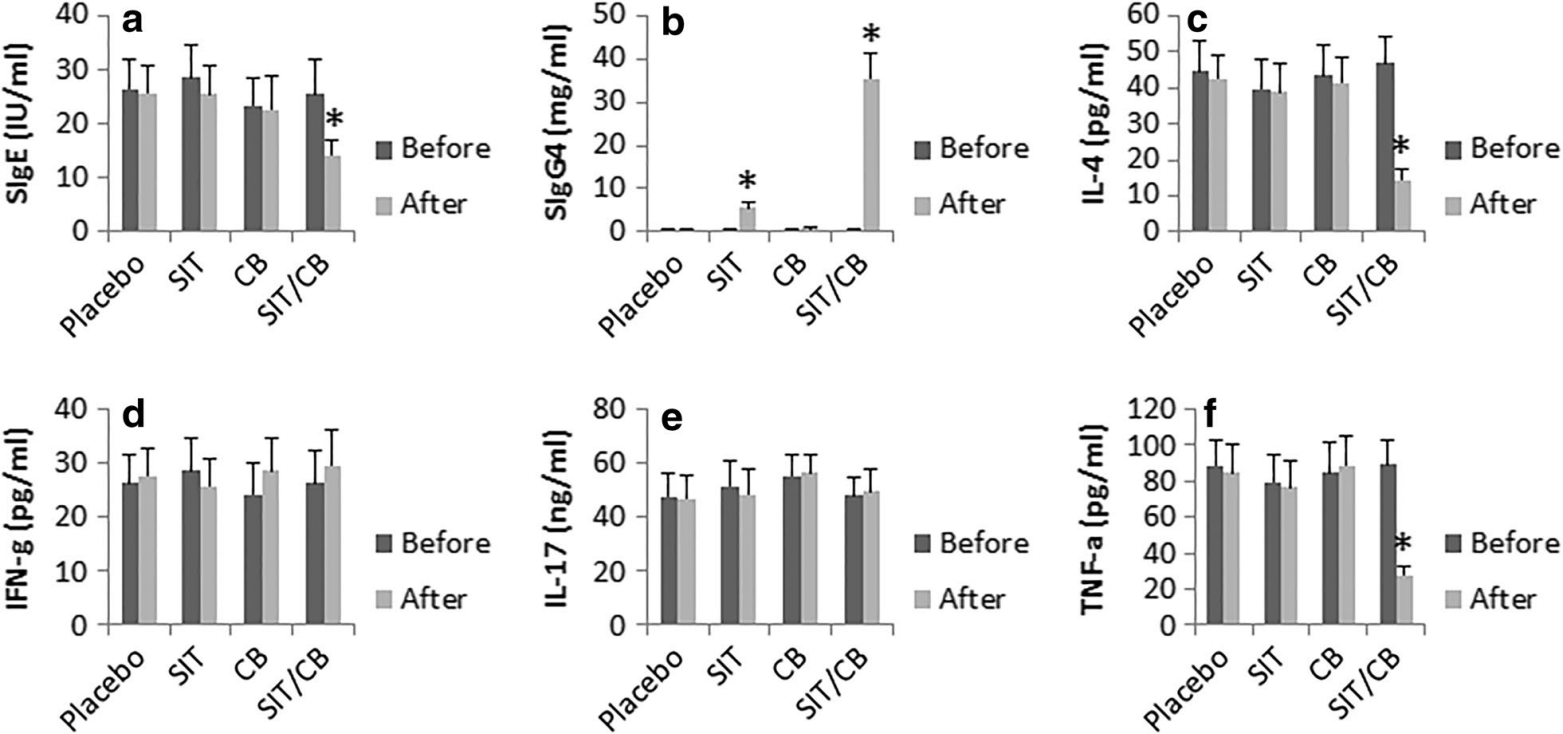
Effect of the therapy on serum IgE, IgG4 and CD4^+^ T cell cytokines. Peripheral blood samples were collected from each patient before and after the therapy. The sera were analyzed by ELISA. The bars indicate the serum levels of SIgE (**a**), SIgG4 (**b**), IL-4 (**c**), IFN-g (**d**), IL-17 (**e**) and TNF-alpha (**f**). The data are presented as mean ± SD. *p < 0.01, compared to the “before” values

The blood samples were collected from each patient before and after the therapy, and analyzed by ELISA. The results showed that the antigen-specific IgE, but not IgG4, IL-4, IFN-γ, IL-17 and TNF-α were detected in all the IBD patients with food allergy. After the therapy, patients treated with SIT/CB showed significantly lower levels of antigen-specific IgE, higher levels of antigen-specific IgG4, lower levels of IL-4 and TNF-α; the levels of IFN-γ and IL-17 were not changed significantly. In those patients treated with placebo, SIT alone or CB alone, all the six parameters remained the same as before the therapy (Fig. 3).

## Discussion

IBD has had a tremendous negative impact on human health and social economy. The current therapies for IBD are not satisfactory. Thus, to develop novel therapeutic strategies is of significance. To this end, we developed a novel treating strategy for a fraction of IBD patients. We treated IBD patients with food allergy with SIT and CB. The results showed profound improvement of IBD clinical symptoms, including reducing the truncated Mayo scores and medication scores, as well as reduced the serum levels of antigen-specific IgE, IL-4, IL-17, TNF-α, and fecal calprotectin while those treated with either SIT alone or CB alone did not induce appreciable improvement.

The association between food allergy and the pathogenesis of IBD has been noted for a long time. Levo et al. [14] in 1980s observed high levels of specific IgE in IBD patients and suggested an association between food allergy and IBD. Joachime [15] noted that people with IBD reported that some foods made them feel better and some foods made them feel worse. Cashman [16] in a review paper proposed that nutrition was an important etiological factor for IBD. Our data are in line with these reports by showing high levels of specific IgE in a group of IBD patients with food allergy. The clinical data have been supported by data from animal model study [3].

It is proposed that multiple factors contribute to the pathogenesis of IBD. Food allergy is one of the major factors in the etiology of IBD [17]. The present data showed that the high levels of specific IgE, IL-4 and TNF-α in the IBD patients with food allergy were down regulated after the therapy of SIT and CB, while the levels of antigen-specific IgG4 were up regulated. The data implicate that besides food allergy, other unknown factors may also exist in these patients to contribute to the pathogenesis of IBD. To up regulate the levels of antigen-specific IgG4 was observed in SIT in previous reports [18]. IgG4 may neutralize IgE [19, 20] and block mast cell activation [21]. Our data show that the serum levels of IgE and TNF-α were suppressed in the patients with IBD and food allergy after the treatment with SIT and CB, indicating that the therapy also blocked mast cell activation since mast cells are one of the major sources of TNF-α in the body [22].

Since patients are being treated for food allergy, the results maybe confounded if improvement was due to food allergy rather than treatment of inflammation in patients with both. In other words, the data show that treating with only SIT did not improve outcome in these patients, how to explain the combination of CB with SIT was truly treating IBD or if it was having a greater impact in food allergy related diarrhea. The data suggest that SIT and CB act synergistically in the generation of the therapeutic effect. Our another study showed that in a food allergy animal model study, the histone deacetylase (HDAC)1 levels in B cells were up regulated. HDAC1 suppressed the expression of IL-10 and facilitated the expression of IgE. Employing the HDAC inhibitor feature, CB facilitated SIT to generate regulatory B cells via releasing butyrate, which is an inhibitor of HDAC1. The induced regulatory B cells fulfilled the immune suppressor function in the inhibition of the allergic inflammation [23]. Others also reported that administration of SIT and CB inhibited asthma clinical symptoms [24].

In summary, combination of SIT and CB efficiently inhibited the clinical symptoms of IBD patients with food allergy.

## Abbreviations

IBD: inflammatory bowel disease
CD: Crohn’s disease
UC: ulcerative colitis
SIT: specific immunotherapy
CB: *Clostridium butyricum*
IL: interleukin
TNF: tumor necrosis factor
